# Supplementary material for: Momentum dependent dxz/yz band splitting in LaFeAsO
Source: Sci Rep. 2020 Nov 9;10:19377. doi: 10.1038/s41598-020-75600-w (PMC7652889; doi:10.1038/s41598-020-75600-w)
Supplement: Supplementary file 1 — Supplementary information. [file 41598_2020_75600_MOESM1_ESM.pdf]

Momentum dependent $d_{xz/yz}$ band splitting in LaFeAsO

S. S. Huh,[1, 2] Y. S. Kim,[1, 2] W. S. Kyung,[1, 2] J. K. Jung,[1, 2] R. Kappenberger,[3] S. Aswartham,[3] B. Büchner,[3, 4] J. M. Ok,[5, 6] J. S. Kim,[5, 6] C. Dong,[7, 8] J. P. Hu,[7, 8] S. H. Cho,[9] D. W. Shen,[9] J. D. Denlinger,[10] Y. K. Kim,[11, 12] and C. Kim[1, 2, *].

[1] Center for Correlated Electron Systems, Institute for Basic Science (IBS), Seoul 08826, Republic of Korea

[2] Department of Physics and Astronomy, Seoul National University (SNU), Seoul 08826, Republic of Korea

[3] Leibniz Institute for Solid State and Materials Research, IFW-Dresden, 01069 Dresden, Germany

[4] 4Institute of Solid State Physics, TU Dresden, 01069 Dresden, Germany

[5] Center for Artificial Low Dimensional Electronic Systems, Institute of Basic Science, Pohang 790-784, Korea

[6] Department of Physics, Pohang University of Science and Technology, Pohang 790-784, Korea

[7] Beijing National Laboratory for Condensed Matter Physics, and Institute of Physics, Chinese Academy of Sciences, Beijing 100190, China

[8] Collaborative Innovation Center of Quantum Matter, Beijing, China

[9] State Key Laboratory of Functional Materials for Informatics, Shanghai Institute of Microsystem and Information Technology (SIMIT), Chinese Academy of Sciences, Shanghai 200050, People's Republic of China

[10] Advanced Light Source, Lawrence Berkeley National Laboratory, California 94720, USA

[11] Department of Physics, Korea Advanced Institute of Science and Technology, Daejeon 34141, Republic of Korea

[12] Graduate school of Nanoscience and Technology, Korea Advanced Institute of Science and Technology, Daejeon 34141, Republic of Korea

Supplementary Note 1: Band calculation

Figure S1 shows the band calculation results of LaFeAsO. The tight binding model described by equation (1),

$$H_t = \sum_{\sigma ij\alpha\beta} t_{ij}^{\alpha\beta} d_{i\alpha\sigma}^{\dagger} d_{j\beta\sigma} + \sum_{\sigma i\alpha} \varepsilon_\alpha d_{i\alpha\sigma}^{\dagger} d_{i\alpha\sigma} \qquad (1)$$

where ε_α is the on-site energy of electron in orbital α = (1, 2, 3, 4, 5) ≡ (xy, x^2-y^2, xz, yz, z^2) and $t_{ij}^{\alpha\beta}$ is the electron hopping integral between sites (i, j) and orbitals (α, β). The parameters were adopted from previous LaFeAsO tight binding model study [1]. To understand nematic phase ARPES result, we adopted an additional term [2] described by,

$$H = g_3 \sum_{k\sigma} \beta_k \left(d_{xz,k,\sigma}^{\dagger} d_{xz,k,\sigma} + d_{yz,k,\sigma}^{\dagger} d_{yz,k,\sigma}\right) \qquad (2)$$

where $\beta_k = \cos k_x - \cos k_y$. Note that our calculation is done without spin-orbit coupling.

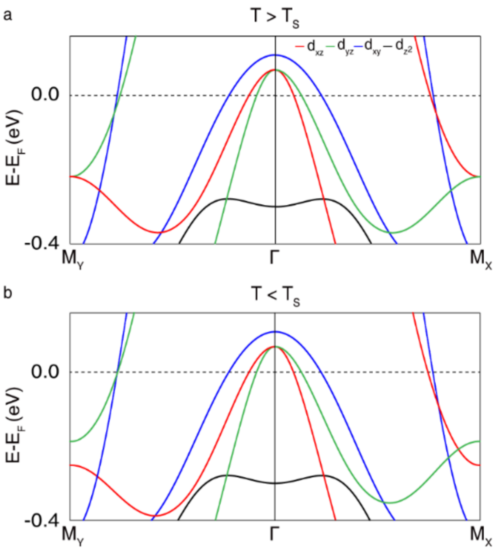

Supplementary Figure 1. Calculated band dispersion (a) above and (b) below T_S. The red, green, blue and black solid lines represent d_{xz}, d_{yz}, d_{xy} and d_{z2} band dispersions, respectively.

Figures S1 (a) and (b) are band dispersions above and below T_S, respectively.

Supplementary Note 2: More energy distribution curves (EDCs) of second derivative near the Γ point.

Figures S2 provides additional details on energy distribution curves (EDCs) analysis of the second derivative data near the Γ point. Figure S2 (a) shows second derivative ARPES data. We plot the temperature dependent EDCs at different momenta in Figure S2 (b). The black arrows indicate the peak positions of $d_{xz/yz}$ band and show temperature independent band splitting behavior through different momenta near the Γ point.

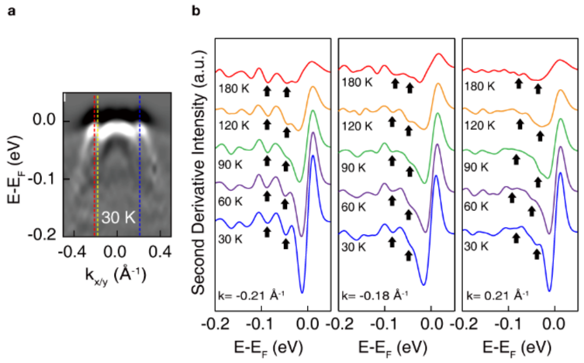

Supplementary Figure 2. (a) Second derivative ARPES data near the Γ point. (b) Temperature dependent energy distribution curves (EDCs) at k= -0.21, -0.18 and 0.21 $Å^{-1}$, indicated by the red, yellow and blue lines in Fig. S2 (a), respectively.

Supplementary References

[1] Graser, S., Maier, T. A., Hirschfeld, P. J. & Scalapino, D. J. Near-degeneracy of several pairing channels in multiorbital models for the Fe pnictides. *New J. Phys.* ***11***, 025016 (2009)

[2] Jiang, K., Hu, J., Ding, H. & Wang, Interatomic Coulomb interaction and electron nematic bond order in FeSe *Phys. Rev. B* ***93***, 115138 (2016).
